# The relationship between penetrative actions and football match outcomes: evidence from the 2022 FIFA World Cup

**DOI:** 10.5114/biolsport.2026.161107

**Published:** 2026-07-06

**Authors:** Tao Yang, Liangzhu Feng, Dingmeng Ren, Zhe Cheng, Xiao Chen, Yushan Liu, Hongyou Liu

**Affiliations:** 1 China Football College, Beijing Sport University, Beijing, China; 2 School of Physical Education, North Minzu University, Yinchuan, China; 3 Research Center of Sports Performance Analysis, School of Physical Education & Sports Science, South China Normal University, Guangzhou, China; 4 School of Football, Wuhan Sports University, Wuhan, China

**Keywords:** FIFA World Cup, Performance Analysis, Winning Probability, Defensive Line Breaks, Penetrative Actions

## Abstract

Penetrative actions play a crucial role in determining success in football. The aim of this study was to quantify the association between penetrative actions and match outcomes at the 2022 FIFA World Cup. Statistical analyses were conducted on 59 matches decided within regular time. The generalised linear mixed model (GLMM) and magnitude-based inference (MBI) were employed to examine the association between various penetrative action parameters and match outcomes (win/draw/loss) at both between- and within-team levels. The results showed that differences in penetrative actions between teams did not yield clear differences in winning probability (all likelihood effects < 95%). However, a two-within-team standard deviation (SD) increased in (i) attempted line breaks, (ii) completed line breaks, (iii) attempted line-breaking passes, (iv) attempted over-the-top line breaks, and (v) completed over-the-top line breaks raised a team’s probability of winning by 34.3% (90% CI ± 15.1%), 30.8% (± 14.6%), 33.8% (± 15.1%), 34.8% (± 15.0%), and 38.2% (± 15.5%), respectively. When playing against opponents utilizing a three-line defensive structure, a two-within-team SD increased in (i) attempted line breaks, (ii) attempted line breaks within the opponent’s formation, (iii) attempted final defensive line breaks, (iv) attempted second defensive line breaks, and (v) completed second defensive line breaks elevated the winning probability by 34.0% (± 14.4%), 32.0% (± 14.6%), 29.4% (± 14.6%), 37.9% (± 14.5%), and 33.3% (± 14.4%), respectively. It is concluded that penetrative actions demonstrate a clear positive association with within-team winning probability. Different types of penetrative actions were associated with different levels of winning probability. The association between penetrative actions and winning probability varies across tactical contexts. These findings provide practicable insights for optimising offensive strategies in elite football.

## INTRODUCTION

Football performance analysis is grounded in a scientific understanding of the game’s unique dynamics and underlying principles [1]. In recent years, the integration of advanced technologies has substantially enhanced this field, enabling analysts to uncover subtle strengths and weaknesses within teams. Such approaches also facilitate the systematic detection of tactical vulnerabilities in opponents [2–4]. These insights not only inform more targeted tactical planning and individualised training programs but also become standard practice among football teams, regardless of competitive level [5–8].

A growing body of research has examined technical and tactical performance indicators in football through descriptive, comparative, and predictive approaches. These studies typically categorise performance metrics into dimensions such as shooting, attacking, and defensive actions [9–11]. In addition, some studies have examined specific offensive behaviours, including passing, ball control, shooting, and goal-scoring opportunities. However, penetrative actions, a fundamental component of attacking play, have received relatively limited attention [12–15].

The limited existing literature on penetrative actions reveals several noteworthy findings. Penetrative passes tend to occur primarily in the half-spaces on both sides of midfield and cluster temporally between the 20^th^ and 25^th^ minutes of match, rather than being evenly distributed across time and space [16]. The duration of opportunities for penetrative passes is shorter compared to supportive or backward passes [17]. Regardless of the defensive conditions or whether the match is played at home or away, penetrative passes have been proven to be more effective than non-penetrative passes [18–20]. These studies, while valuable, remain largely descriptive and comparative, focusing on when and where penetrative actions occur rather than why or how they influence match outcomes. This limitation stems largely from the absence of comprehensive, context-rich datasets of penetration actions in earlier research, which has restricted efforts to elucidate the extent to which penetrative actions contribute to match success.

In response to this limitation, FIFA launched the “FIFA Football Language” project to standardise match analysis globally through an integration of expert-driven and data-based approaches. Implemented during the 2022 FIFA World Cup, it generated over 15,000 contextualised data points per match and, for the first time, systematically defined and recorded detailed penetrative action parameters [21]. This data has been used in research in recent years [22–26]. This innovation offers the data depth and contextual precision required to rigorously examine the contribution of penetrative actions to match success.

Building upon these advancements, the present study seeks to address a key gap in the existing literature by providing empirical evidence on the relationship between penetrative actions and match outcomes in elite football. Leveraging the high-resolution dataset from the 2022 FIFA World Cup and employing advanced statistical and modelling approaches, this study attempts to move beyond descriptive analyses to quantify the marginal association of penetrative actions with team success. By examining both the type and tactical context of these actions, the research aims to clarify their functional role within attacking dynamics and to inform evidence-based tactical decision-making. Specifically, the study is guided by the following hypotheses:

(1) penetrative actions are positively associated with match outcomes.
(2) different types of penetrative actions demonstrate varying degrees of association with match outcomes.
(3) the association between penetrative actions and match outcomes varies across different tactical contexts.

## MATERIALS AND METHODS

### Sample and variables

The sample of this study comprises performance data from 59 matches of the 2022 FIFA World Cup in Qatar, excluding matches that went into extra time. The data was sourced from FIFA’s official match reports and is freely accessible on the internet via the following link: https://www.fifatrainingcentre.com/en/fwc2022/post-match-summaries/post-matchsummary-reports.php. The data from these matches were encoded and calculated by a tracking system (multi-camera optical tracking system: TRACAB Gen^®^, chyronhego), the validity and accuracy of data collection by TRACAB Gen^®^ were previously documented [27]. This database has recently been used in similar research [22–26]. As this data is freely available to the general public, no ethical approval was required. The study includes 39 penetrative action parameters [21], with their definitions and descriptions provided in Table 1. All parameter values were standardised to reflect the scenario under average possession conditions. The standardised value of a penetrative action for team i in match j (*P A*_*std,i,j*_) was computed as:

$$PA_{std,i,j}=PA_{raw,i,j}\times \frac{P_{i,j}}{\bar{P}}$$ #(1)

where *P A_raw,i,j_* denotes the raw value of the penetrative action for team i in match j; *P*_i,j_ represents the possession rate (%) of team i in match j; $\bar{P}$ is the average possession rate across all teams and matches.

**TABLE 1:** Definitions of penetrative action parameters

Parameter	Definition
**Undifferentiated tactical situations**	

Attempted line breaks	The number of attempts to penetrate a defensive line composed of at least two opposing players through passing or dribbling.
Completed line breaks	The number of successful instances where the ball penetrate a defensive line composed of at least two opposing players through passing or dribbling.
Completed final defensive line breaks	The number of successful instances where the ball penetrate the final defensive line composed of at least two opposing players through passing or dribbling.
Attempted line-breaking passes	The number of attempts to penetrate a defensive line through passing (excluding crosses).
Attempted line-breaking crosses	The number of attempts to penetrate a defensive line through crosses.
Attempted line-breaking dribbles	The number of attempts to penetrate a defensive line through dribble.
Attempted low-pass line breaks	The number of attempts to penetrate a defensive line through passes or dribbles between the two outermost defenders, with the ball at a height below head level.
Completed low-pass line breaks	The number of successful passes or dribbles to penetrate a defensive line between the two outermost defenders, with the ball at a height below head level.
Attempted line break bypass	The number of attempts to penetrate a defensive line through passes or dribbles around the two outermost defenders
Completed line break bypass	The number of successful passes or dribbles to penetrate a defensive line around the two outermost defenders.
Attempted over-the-top line breaks	The number of attempts to penetrate a defensive line through passes above the formation, with the ball above head height.
Completed over-the-top line breaks	The number of successful passes to penetrate a defensive line above the formation, with the ball above head height.

**Against Four Defensive Lines**	

Attempted line breaks	The number of attempts to penetrate a defensive line composed of at least two opposing players through passing or dribbling.
Attempted line breaks within the opponent’s formation	The number of attempted passes that penetrate a defensive line (composed of at least two players) to find a teammate within the opponent’s formation.
Attempted line breaks outside the opponent’s formation	The number of attempted passes that penetrate a defensive line (composed of at least two players) to find a teammate outside the opponent’s formation.
Attempted final defensive line breaks	The number of attempts to penetrate the final defensive line when facing four defensive lines.
Completed final defensive line breaks	The number of successful passes or dribbles to penetrate the final defensive line when facing four defensive lines.
Attempted third defensive line breaks	The number of attempts to penetrate the third defensive line when facing four defensive lines.
Completed third defensive line breaks	The number of successful passes or dribbles to penetrate the third defensive line when facing four defensive lines.
Attempted second defensive line breaks	The number of attempts to penetrate the second defensive line when facing four defensive lines.
Completed second defensive line breaks	The number of successful passes or dribbles to penetrate the second defensive line when facing four defensive lines.
Attempted first defensive line breaks	The number of attempts to penetrate the first defensive line when facing four defensive lines.
Completed first defensive line breaks	The number of successful passes or dribbles to penetrate the first defensive line when facing four defensive lines.

**Against Three Defensive Lines**	

Attempted line breaks	The number of attempts to penetrate a defensive line composed of at least two opposing players through passing or dribbling.
Attempted line breaks within the opponent’s formation	The number of attempted passes that penetrate a defensive line (composed of at least two players) to find a teammate within the opponent’s formation.
Attempted line breaks outside the opponent’s formation	The number of attempted passes that penetrate a defensive line (composed of at least two players) to find a teammate outside the opponent’s formation.
Attempted final defensive line breaks	The number of attempts to penetrate the final defensive line when facing three defensive lines.
Completed final defensive line breaks	The number of successful passes or dribbles to penetrate the final defensive line when facing three defensive lines.
Attempted second defensive line breaks	The number of attempts to penetrate the second defensive line when facing three defensive lines.
Completed second defensive line breaks	The number of successful passes or dribbles to penetrate the second defensive line when facing three defensive lines.
Attempted first defensive line breaks	The number of attempts to penetrate the first defensive line when facing three defensive lines.
Completed first defensive line breaks	The number of successful passes or dribbles to penetrate the first defensive line when facing three defensive lines.

**Against Two Defensive Line**s	

Attempted line breaks	The number of attempts to penetrate a defensive line composed of at least two opposing players through passing or dribbling.
Attempted line breaks within the opponent’s formation	The number of attempted passes that penetrate a defensive line (composed of at least two players) to find a teammate within the opponent’s formation.
Attempted line breaks outside the opponent’s formation	The number of attempted passes that penetrate a defensive line (composed of at least two players) to find a teammate outside the opponent’s formation.
Attempted final defensive line breaks	The number of attempts to penetrate the final defensive line when facing two defensive lines.
Completed final defensive line breaks	The number of successful passes or dribbles to penetrate the final defensive line when facing two defensive lines.
Attempted first defensive line breaks	The number of attempts to penetrate the first defensive line when facing two defensive lines.
Completed first defensive line breaks	The number of successful passes or dribbles to penetrate the first defensive line when facing two defensive lines.

### Procedure and statistical analysis

The dataset was imported into SAS^®^ Studio for statistical analysis. A generalised linear mixed model (GLMM) was implemented using Proc Glimmix. Each standardised penetrative action parameter was treated as a predictor variable (main effect), while the odds of winning were set as the outcome variable.

A total of 39 independent multivariable logistic regression models were established. To account for repeated measures within teams, team identity was included as a random effect, ensuring proper handling of matches played by the same team. Match outcomes were coded as 3/1/0, representing win, draw, and loss, respectively. The GLMM estimated two key effects: (1) Within-team change: reflects how changes in a specific penetrative action parameter within the same team (increase or decrease) influence its probability of winning. The final reported value represents the average effect across all 32 teams. (2) Between-team difference: measures how differences in a specific penetrative action parameter across different teams disdinguished their probability of winning. The non-clinical magnitude-based inference (MBI) was used for further interpretation of the effects. Specifically, two scenarios were evaluated: The impact of increasing a given parameter by two within-team standard deviations (SD) on a team’s winning probability. The difference in winning probability between a team with a parameter value of one-between-team SD above the mean and a team with a value of one SD below the mean. For both scenarios, 90% confidence intervals (CI) were calculated. Within-team SD represents a team’s match-to-match variability and was estimated from model residuals. Between-team SD reflects the variation in average values across different teams and was derived from the random effects of the model [28].

In football performance analysis, a 10% change/difference in winning probability was set as the smallest worthwhile effect [28]. A parameter was deemed to have a clear association with winning probability if its 90% CI did not simultaneously include both positive and negative smallest worthwhile effect (± 10%). The likelihood of a clear positive, negative, or trivial effect was categorised as follows: < 0.5% most unlikely, 0.5–5% very unlikely, 5–25% unlikely, 25–75% possibly, 75–95% likely, 95–99.5% very likely, > 99.5% most likely [29]. To reduce the risk of Type II errors, only effects with a probability of greater than 95% were analysed and discussed.

## RESULTS

Table 2 presents the descriptive statistics for each penetrative action parameter across matches where teams won, drew, or lost in the 2022 FIFA World Cup. Figure 1 shows the between- and within-team relationships of penetration action parameters and the winning probability. As has been shown, the between-team differences in penetrative actions did not result in clear winning probability differences (all likelihoods < 95%). However, increasing the number of attempted line breaks, completed line breaks, attempted line-breaking passes, attempted over-the-top line breaks, and completed over-the-top line breaks by two within-team SD could increase a team’s probability of winning by 30.8%–38.2% (≥ 95% likelihood). When facing three defensive lines of the opponent, increasing by two within-team SD in the number of attempted line breaks, attempted line breaks within the opponent’s formation, attempted final defensive line breaks, attempted second defensive line breaks, and completed second defensive line breaks could raise a team’s win probability by 29.4%–37.9% (≥ 95% likelihood).

**Table 2:** Descriptive data of penetrative action parameters (Mean ± SD)

	Raw Value			Standardised Value		

	Win	Draw	Loss	Win	Draw	Loss
**Undifferentiated tactical situations**						

Attempted line breaks	130 ± 27	132 ± 19	129 ± 26	141 ± 30	135 ± 20	130 ± 27
Completed line breaks	94 ± 29	92 ± 23	90 ± 24	98 ± 16	93 ± 16	89 ± 16
Completed final defensive line breaks	10.6 ± 5.2	9.7 ± 4.6	8.9 ± 5.8	11.1 ± 4.6	9.5 ± 3.8	8.4 ± 4.4
Attempted line-breaking passes	120 ± 25	123 ± 18	120 ± 25	130 ± 29	126 ± 19	121 ± 26
Attempted line-breaking crosses	3.6 ± 2.0	3.4 ± 2.0	4.1 ± 2.5	3.9 ± 2.3	3.3 ± 1.8	4.1 ± 2.2
Attempted line-breaking dribbles	6.1 ± 2.9	5.2 ± 3.3	5.6 ± 2.7	6.6 ± 3.3	5.4 ± 3.5	5.6 ± 2.7
Attempted low-pass line breaks	34.7 ± 14.2	36.0 ± 11.8	36.0 ± 11.4	36.7 ± 12.2	36.2 ± 9.9	35.7 ± 10.3
Completed low-pass line breaks	28.3 ± 12.8	28.7 ± 10.2	28.2 ± 9.7	29.4 ± 9.0	28.7 ± 8.3	27.9 ± 8.7
Attempted line break bypass	47 ± 20	45 ± 18	45 ± 18	48 ± 11	45 ± 14	43 ± 10
Completed line break bypass	41.8 ± 19.1	40.3 ± 16.4	39.4 ± 16.9	42.0 ± 10.8	39.6 ± 12.8	37.3 ± 9.5
Attempted over-the-top line breaks	48.1 ± 12.4	50.6 ± 11.6	48.7 ± 12.4	56.1 ± 27.3	53.6 ± 19.8	52.0 ± 22.6
Completed over-the-top line breaks	23.1 ± 6.2	23.4 ± 5.9	22.3 ± 7.3	26.4 ± 12.1	24.5 ± 8.7	23.7 ± 10.8

**Against Four Defensive Lines**						

Attempted line breaks	6.4 ± 3.1	6.8 ± 3.3	6.7 ± 3.3	7.5 ± 4.6	6.9 ± 3.2	7.4 ± 4.9
Attempted line breaks within the opponent’s formation	4.1 ± 2.0	4.0 ± 2.0	4.4 ± 2.6	4.9 ± 3.1	4.1 ± 2.1	4.9 ± 3.3
Attempted line breaks outside the opponent’s formation	2.2 ± 1.8	2.8 ± 1.9	2.2 ± 1.8	2.6 ± 2.4	2.8 ± 1.8	2.5 ± 2.4
Attempted final defensive line breaks	1.4 ± 1.2	1.4 ± 1.0	1.3 ± 1.2	1.8 ± 1.7	1.4 ± 1.2	1.5 ± 1.6
Completed final defensive line breaks	0.47 ± 0.74	0.65 ± 0.67	0.37 ± 0.78	0.52 ± 0.90	0.63 ± 0.70	0.38 ± 0.81
Attempted third defensive line breaks	2.0 ± 1.6	2.0 ± 1.6	1.9 ± 1.7	2.2 ± 1.9	2.0 ± 1.7	2.2 ± 2.2
Completed third defensive line breaks	1.0 ± 1.2	0.8 ± 1.2	0.9 ± 1.0	1.1 ± 1.4	0.8 ± 1.2	1.0 ± 1.2
Attempted second defensive line breaks	1.8 ± 1.6	2.1 ± 1.2	2.2 ± 1.8	2.1 ± 2.0	2.2 ± 1.4	2.4 ± 2.8
Completed second defensive line breaks	1.1 ± 1.4	1.4 ± 1.0	1.2 ± 1.1	1.4 ± 1.7	1.5 ± 1.2	1.3 ± 1.5
Attempted first defensive line breaks	1.2 ± 1.2	1.4 ± 1.4	1.2 ± 1.1	1.3 ± 1.4	1.3 ± 1.2	1.2 ± 1.2
Completed first defensive line breaks	1.1 ± 1.1	1.4 ± 1.4	1.0 ± 1.1	1.2 ± 1.3	1.3 ± 1.2	1.0 ± 1.1

**Against Three Defensive Lines**						

Attempted line breaks	84 ± 22	89 ± 17	81 ± 20	91 ± 26	91 ± 19	83 ± 25
Attempted line breaks within the opponent’s formation	47.8 ± 11.9	51.3 ± 9.6	45.9 ± 11.7	54.3 ± 21.5	53.8 ± 16.6	48.5 ± 18.4
Attempted line breaks outside the opponent’s formation	35.7 ± 15.2	37.8 ± 12.8	34.9 ± 12.5	36.9 ± 10.0	37.6 ± 10.0	34.7 ± 10.4
Attempted final defensive line breaks	10.2 ± 4.5	10.5 ± 2.6	9.0 ± 3.7	11.1 ± 4.7	10.7 ± 2.9	8.9 ± 3.3
Completed final defensive line breaks	5.2 ± 3.1	4.4 ± 2.4	4.1 ± 2.9	5.4 ± 2.9	4.2 ± 1.9	4.0 ± 2.5
Attempted second defensive line breaks	38.0 ± 10.4	39.9 ± 8.7	35.1 ± 11.6	42.9 ± 17.9	41.4 ± 11.4	36.8 ± 15.1
Completed second defensive line breaks	23.6 ± 7.7	24.7 ± 7.3	21.6 ± 9.0	25.8 ± 9.7	25.2 ± 7.1	22.0 ± 9.7
Attempted first defensive line breaks	35.3 ± 14.4	38.7 ± 10.7	36.6 ± 10.2	37.2 ± 11.5	39.3 ± 9.7	37.5 ± 11.1
Completed first defensive line breaks	31.1 ± 14.7	32.9 ± 10.6	31.2 ± 10.0	32.3 ± 10.7	33.0 ± 8.9	31.4 ± 8.8

**Against Two Defensive Lines**						

Attempted line breaks	40 ± 19	48 ± 50	42 ± 20	42 ± 17	52 ± 69	40 ± 13
Attempted line breaks within the opponent’s formation	23.1 ± 9.5	22.2 ± 5.7	24.5 ± 11.1	25.2 ± 11.2	22.5 ± 5.2	23.7 ± 8.7
Attempted line breaks outside the opponent’s formation	17.0 ± 10.8	13.9 ± 6.5	17.3 ± 10.6	16.9 ± 8.0	13.6 ± 4.8	16.0 ± 6.2
Attempted final defensive line breaks	7.0 ± 4.6	7.2 ± 3.8	6.5 ± 4.1	7.6 ± 4.7	7.1 ± 3.3	6.1 ± 3.2
Completed final defensive line breaks	4.8 ± 3.8	4.7 ± 2.7	4.4 ± 3.6	5.1 ± 3.8	4.7 ± 2.4	4.1 ± 2.7
Attempted first defensive line breaks	32.6 ± 16.1	29.0 ± 9.1	35.3 ± 17.5	34.0 ± 13.7	29.1 ± 7.4	33.6 ± 11.0
Completed first defensive line breaks	25.3 ± 14.0	21.6 ± 7.5	25.2 ± 13.9	25.5 ± 9.7	21.4 ± 5.0	23.8 ± 8.8

**FIG. 1:**
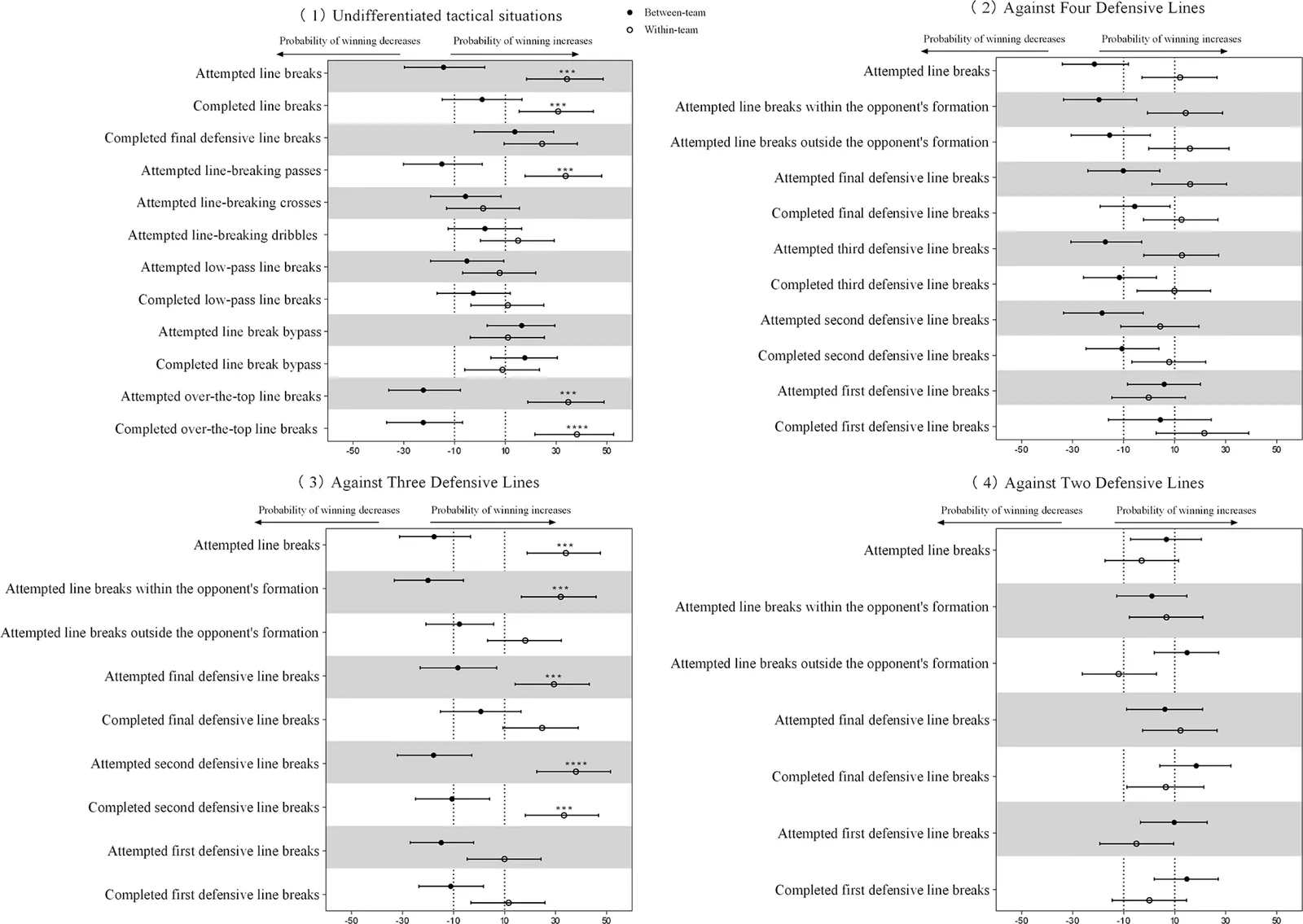
Relationship between penetrative action parameters and winning probability in the 2022 FIFA World Cup matches. Solid black dots represent the difference in team winning probability when there is a two between-team standard deviation difference in the parameter values. Hollow black dots indicate the change in winning probability when the parameter values increase by two within-team standard deviations. Error bars represent the 90% confidence interval for the difference/change values. Asterisks denote the likelihood of the effect: *** very likely, **** most likely.

## DISCUSSION

This study explored and quantified the relationship between penetrative actions and football match outcomes. The results revealed a clear discrepancy between within-team and between-team effects. Specifically, increases in penetrative actions within the same team from match to match were associated with higher probabilities of winning, whereas teams that generally performed more penetrative actions than others did not demonstrate a higher overall winning probability across the tournament. This finding suggests that penetrative actions are more relevant for explaining variations in the performance of the same team across matches rather than for distinguishing stronger teams from weaker teams in a tournament context. In other words, penetrative actions appear to operate primarily as context-dependent and match-specific tactical behaviours rather than stable team-level advantages. These findings enhance the understanding of penetrative actions in football and provide a theoretical basis for training and match preparation in real-world settings.

### Impact under undifferentiated tactical solutions

Among all situational penetrative metrics, both attempted and completed line breaks exerted a clear positive effect on match-winning probability, confirming the offensive effectiveness of the penetration principle. This principle emphasises bypassing the opponent’s defensive structure to disrupt their organization, generate numerical or positional advantages, and create higher-quality scoring opportunities [19, 22, 30–32]. Previous studies have identified through balls and forward passes as cardinal parameters of penetrative play. A through pass is a classic example of a penetrative action, referring in this study to the number of passes that break through the final defensive line. Research has demonstrated that the frequency of through balls is a key metric for distinguishing between match outcomes, qualifying and non-qualifying teams, and strong and weak teams, findings that are consistent with the results of the present study [6, 33–35]. There are two distinct perspectives in the research on forward passing. The first perspective suggests positive effects of forward passing: a higher proportion of forward passes prior to scoring is associated with improved scoring efficiency [36]; strong teams demonstrate a greater proportion of forward passes in central areas compared to weaker teams [37]; and higher-level teams achieve greater passing accuracy and a higher proportion of forward passes when breaking through the opponent’s defense [38]. Conversely, some studies report that teams that lose tend to make more forward passes than those that win or draw. In closely contested matches, forward passing may negatively affect the likelihood of winning, as teams eager to score often adopt a more aggressive and direct playing style. When facing opposition’s high defensive pressure, this approach can result in an increased frequency of indiscriminate and low-quality passes, leading to more errors [39]. One possible explanation for these apparently contradictory findings is that previous studies primarily evaluated passing direction rather than its penetrative effectiveness. Earlier statistical approaches often overlooked the penetrative dimension of passing and focused solely on whether passes were played forward. However, assessing passing effectiveness should consider both the opponent’s defensive structure and the extent to which passes break defensive lines [30, 40–42]. Moreover, the success rate and spatial location of forward passes are also critical, especially in the attacking third and the opponent’s penalty area, where effective forward passing enables attackers to face the goal directly and create shooting or scoring opportunities [43, 44].

Among the three types of penetration actions, the only parameter that demonstrates a clear positive effect on the probability of winning is the number of attempted line-breaking passes. This finding is generally consistent with previous research. Hughes and Franks demonstrated that penetrative passes are more effective than nonpenetrative passes [45]. Tenga et al. further showed that the frequency of penetrative passes is positively correlated with the creation of scoring opportunities [46]. Rein et al., in their research on passing efficiency, evaluated effectiveness by considering both the spatial advantage between the ball carrier and the goal and the number of defenders overcome [30]. They found that both dimensions, spatial advantage and the number of defenders surpassed, positively influence the number of goals scored and the probability of winning. Together, these findings suggest that the effectiveness of passing actions in football should be evaluated not only by their direction but also by their ability to penetrate defensive structures. These results corroborate the findings of the present study.

Among the three different directions of penetrative actions, both the frequency of attempted over-the-top line breaks and the number of completed over-the-top line breaks positively affect the probability of winning. Over-the-top passes compensate for the limitations of some previous studies on penetrating passes, which only provided a two-dimensional perspective [16]. However, to fully understand the distinct positive impact of this specific type of penetration compared to low-pass or bypass, we must consider the factor of opposition interaction. As highlighted by Tenga et al., the effectiveness of creating penetration is intrinsically linked to the degree of defensive balance of the opponent [47]. Although our study did not directly analyze the ‘play-counter play’ dynamics, it is plausible that over-the-top passes are particularly effective because they often exploit moments of defensive imbalance. These passes typically occur when the opponent applies high defensive pressure or maintains a high defensive line, leaving valuable space behind the defenders. In such scenarios, the defense is often in a state of transition, making them vulnerable to direct balls over the top. In contrast, low-pass attempts frequently encounter a structured and balanced defensive block, where the opposition is well-organized to intercept or delay the attack. As a result, over-the-top passes are frequently used as an attacking strategy in direct attacks and counterattacks, particularly when they breach the final defensive line, which poses the greatest threat. This interpretation aligns with findings that the goal-scoring efficiency of direct attacks and counterattacks surpasses that of meticulously organised attacks, primarily because these strategies are designed to capitalize on defensive imbalance [19, 46, 48–50].

### Impact under different tactical situations

An analysis of penetration attempts in different tactical situations revealed that only those made against an opponent’s three defensive lines have a clear positive impact on the probability of winning the match. This finding further supports the idea that the effectiveness of penetrative actions is highly dependent on the specific tactical context of the match.

To interpret this finding, we can also consider the factor of opposition interaction. As suggested by Tenga et al., the outcome of an attack is heavily dependent on the opponent’s degree of defensive balance [47]. Compared to attempts against an opponent’s two defensive lines, which are often designed to minimize space and prioritize structural stability, attempts against an opponent’s three defensive lines may be more susceptible to moments of ‘defensive imbalance’ during rapid transitions. Conversely, when against an opponent’s four defensive lines, the defense typically possesses greater layering and redundancy (e.g., a holding midfielder screen), allowing them to recover balance even if one line is breached. Therefore, the significant impact of penetrating against an opponent’s three defensive lines likely reflects the optimal scenario where the attacking team can exploit the space between lines before the opposition can restore their defensive organization. Furthermore, attempted line breaks within the opponent’s formation have a greater effect on win probability than those outside the opponent’s formation. As explained in previous research, successfully breaking through the defensive lines and into the opponent’s formation can create more shooting and assist opportunities [50]. In terms of the effectiveness of these penetrations, only attempts to break through the final defensive line, the second defensive line, and successful breakthroughs of the second line show a clear positive influence on win probability. This finding supports the research by Seabra et al., who state that while receiving the ball behind the final defensive line is less frequent, it poses the greatest threat to the goal. Additionally, the space between the second and final defensive lines is the primary area for assists, receiving passes, and shots [50].

### Practical implications

The present findings have several practical implications for coaches, analysts, and practitioners in elite football, particularly in relation to the tactical use of penetrative actions during match play. Because penetrative actions were associated with winning probability primarily at the within-team level rather than the between-team level, these actions should not be interpreted as stable indicators of team strength across a tournament. Instead, they should be considered matchspecific tactical behaviours that may enhance a team’s probability of success when effectively executed in a particular match context.

From a practical perspective, coaches should emphasise the situational use of penetrative actions rather than simply increasing their overall frequency across matches. Tactical preparation should therefore focus on identifying moments in which penetrative actions are most likely to disrupt the opponent’s defensive organisation, particularly when exploitable spaces emerge between defensive lines. Furthermore, performance analysts may benefit from integrating opponent scouting more closely with offensive planning, as the effectiveness of penetrative actions appears to depend strongly on the opponent’s defensive organisation and balance. Training design should also incorporate representative game situations that simulate different defensive structures, enabling players to recognise and exploit opportunities for penetration under varying tactical conditions.

### Limitations

This study has certain limitations. First, penetrative action parameters are formally introduced for World Cup matches for the first time. However, because the number of World Cup matches is limited, the sample size precludes analyses across different match contexts, such as match phases, team strength, qualifying and non-qualifying teams, and match strategies. Second, while we categorized penetrative actions by defensive formation (e.g., against three lines), we did not quantify the degree of the opponent’s defensive balance at the moment of the action, which omits an important dimension of opponent interaction. Third, multicollinearity among the 39 penetrative parameters warrants attention because these variables are hierarchically related and overlapping, reflecting multiple contextual expressions of a single latent construct, overall penetrative intensity. Future work should collect larger, multi-context samples and integrate spatiotemporal tracking data to analyze offensive penetration and the corresponding defensive responses simultaneously, yielding a more holistic view of match-play action. In addition, advanced statistical approaches such as dimensionality reduction (e.g., PCA/factor analysis) or multivariate modeling should be employed to extract independent dimensions and estimate their unique contributions while accounting for intercorrelations.

## CONCLUSIONS

The findings of this study provide support for the proposed hypotheses. First, penetrative actions in football are positively associated with match outcomes: teams with higher numbers of attempted line breaks and completed line breaks demonstrate a higher probability of winning. Second, different types of penetrative actions demonstrate varying associations with match outcomes: teams with higher numbers of attempted line-breaking passes, attempted over-the-top line breaks, and completed over-the-top line breaks show a higher probability of winning. Finally, the association between penetrative actions and match outcomes varies across different tactical situations: only when facing three defensive lines are increases in attempted line breaks, attempted line breaks within the opponent’s formation, attempted final defensive line breaks, attempted second defensive line breaks, and completed second defensive line breaks associated with a higher probability of winning. Importantly, the present findings suggest that penetrative actions should not be interpreted as stable indicators of team strength across matches, but rather as context-dependent and match-specific tactical behaviours that may enhance winning probability in particular game situations.

From a practical perspective, these findings provide useful insights for coaches and performance analysts in elite football. Instead of simply increasing the overall frequency of penetrative actions, tactical preparation should emphasise the situational use of these actions, particularly in moments when spaces emerge between defensive lines. In addition, training design may benefit from incorporating representative match scenarios that help players recognise and exploit opportunities to penetrate defensive structures under different tactical conditions.

## Data Availability

The datasets used and/or analysed during the current study are available from the corresponding author upon reasonable request.
